# Bone Metastatic Breast Cancer: Advances in Cell Signaling and Autophagy Related Mechanisms

**DOI:** 10.3390/cancers13174310

**Published:** 2021-08-26

**Authors:** Ahmad Othman, Marcus Winogradzki, Linus Lee, Manish Tandon, Alan Blank, Jitesh Pratap

**Affiliations:** Department of Anatomy & Cell Biology, Cell & Molecular Medicine, Rush University Medical Center, Chicago, IL 60612, USA; Ahmad_H_Othman@rush.edu (A.O.); Marcus_C_Winogradzki@rush.edu (M.W.); Linus_Lee@rush.edu (L.L.); mtandon1.1@gmail.com (M.T.); ALAN_BLANK@rush.edu (A.B.)

**Keywords:** breast cancer, bone metastasis, cell signaling, autophagy, tumor-derived factors, microenvironment-derived factors

## Abstract

**Simple Summary:**

Bone metastasis is a leading cause of breast cancer-related deaths. The interaction between metastatic cancer cells and bone-resident cells promotes tumor growth and bone loss. Metastatic tumors within the bone can contribute to complications including pathological fracture, hypercalcemia, spinal cord compression, and pain. The underlying molecular mechanisms that regulate these interactions in the bone microenvironment are not completely understood. Multiple cell signaling pathways, transcription factors, miRNAs, and secretory factors have been shown to promote bone metastasis. Here, we review the mechanisms by which tumor-derived and tumor-microenvironment-derived factors contribute to bone metastasis. We discuss recent findings highlighting the role of cell signaling and the autophagy pathway in bone metastasis. Furthermore, we discuss the clinical management, treatment options, current challenges, and potential novel targeting strategies of metastatic bone disease.

**Abstract:**

Bone metastasis is a frequent complication of breast cancer with nearly 70% of metastatic breast cancer patients developing bone metastasis during the course of their disease. The bone represents a dynamic microenvironment which provides a fertile soil for disseminated tumor cells, however, the mechanisms which regulate the interactions between a metastatic tumor and the bone microenvironment remain poorly understood. Recent studies indicate that during the metastatic process a bidirectional relationship between metastatic tumor cells and the bone microenvironment begins to develop. Metastatic cells display aberrant expression of genes typically reserved for skeletal development and alter the activity of resident cells within the bone microenvironment to promote tumor development, resulting in the severe bone loss. While transcriptional regulation of the metastatic process has been well established, recent findings from our and other research groups highlight the role of the autophagy and secretory pathways in interactions between resident and tumor cells during bone metastatic tumor growth. These reports show high levels of autophagy-related markers, regulatory factors of the autophagy pathway, and autophagy-mediated secretion of matrix metalloproteinases (MMP’s), receptor activator of nuclear factor kappa B ligand (RANKL), parathyroid hormone related protein (PTHrP), as well as WNT5A in bone metastatic breast cancer cells. In this review, we discuss the recently elucidated mechanisms and their crosstalk with signaling pathways, and potential therapeutic targets for bone metastatic disease.

## 1. Introduction

Metastatic bone disease (MBD) is a significant cause of patient mortality and poses a substantial challenge to clinicians. While strides have been made in the development of screening technologies and effective treatments, MBD prevalence will continue to grow due to gaps in our knowledge of the mechanisms regulating metastatic tumor growth and cellular interactions between metastatic tumors and the local microenvironment. Current estimates show that breast cancer is the most common form of cancer among women within the US, and it projected to account for 15% of all cancers diagnosed, and 7% of cancer related deaths within the US during 2020 [1]. Nearly 30% of breast cancer patients will exhibit distant metastasis, the development of which is associated with a 30% five-year survival rate with a median 2-year survival time. The vast majority of breast cancer deaths, upwards of 90%, are attributed to metastasis [2,3,4,5]. It has been estimated that approximately 70 percent of patients with metastatic breast cancer will develop bone metastasis, with approximately 5–8 percent of patients exhibiting metastatic tumors at the time of diagnosis [3,6,7]. The overall incidence of MBD is rising, from 18.00 to 19.06 per 100,000 from 2010 to 2015 in a SEER study [8]. As novel therapies have improved survival in breast cancer patients, bone metastasis will become an increasingly important consideration. The results of cytotoxic chemotherapy regimens have been important in improving the outcomes of patients with metastatic breast carcinoma but seem to have plateaued in terms of survival improvement within the last decade [9,10]. New targeted therapies are needed in this arena in order to improve patient survival while minimizing additional side effects which affect patient quality of life. Bisphosphonates and denosumab, an antibody against RANKL, have demonstrated reduction of skeletal morbidity and are commonly used as part of the treatment regimen [11,12,13]. These medications are crucial in avoiding pathologic fractures and have been shown to significantly improve patient quality of life [12,14,15].

Metastatic bone disease places an increased burden upon healthcare systems and may be a significant driver of total oncologic expenditure. In 2004, the national cost burden of patients with MBD was estimated at USD 12.6 billion [16]. Not only does presence of MBD increase healthcare costs, treatment of skeletal-related events (SREs) secondary to MBD also requires increased healthcare resource utilization. A 2004 review showed a higher rate of SREs in breast cancer patients compared to prostate cancer patients over a 24-month period [17], which suggests that the overall incidence of breast cancer SREs outnumbers prostate cancer SREs. Consequently, the overall economic burden of treating breast cancer with MBD is potentially quite significant. Prophylactic surgery for impending fractures may be one method to reduce overall cost. One study found that prophylactic surgery cost nearly USD 21,000 less per patient than treatment of a pathologic fracture [18].

## 2. Bone Metastasis

### 2.1. Metastatic Potential and Organ Tropism

The metastatic process can be divided into three stages consisting of (1) detachment of cells from the primary tumor, (2) invasion and migration, and (3) the subsequent extravasation from the vasculature and adhesion within the metastatic site to the development of a secondary tumor. Each stage presents a unique set of metabolic and physical challenges which must be overcome [19,20]. Several animal studies have shown that within 24 h of entry to the circulation only 0.01% of cells are able to complete this process and produce metastatic tumors. It has been suggested that a tumor is composed of a heterogeneous population of cells with varying metastatic potentials, with only a specific subset capable of surviving the three stages mentioned. Using single cell clones of the MDA-MB-231 breast cancer cell line, Gupta and colleagues showed subsets of cells with distinct gene signatures and metastatic profiles [21,22]. While the development of specific organotropic clones has helped to elucidate the mechanisms of metastasis, the dynamic nature of tumor cells such as the acquisition of mutations and the genetic instability can shift the behavior of tumor cells leading to potential metastasis [23].

### 2.2. Disease Onset and Progression

**Figure 1:**
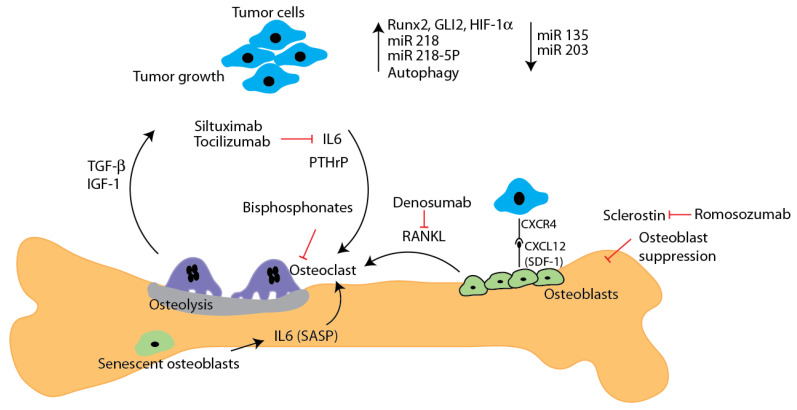
The main factors and pathways contributing to bone metastatic disease are shown. Tumor cell-derived factors such as pro-osteoclast factors (IL6, RANKL, and PTHrP) promote osteoclastic activity and cause severe bone loss.. Osteolysis releases growth factors such as TGF β and IGF-1 further increase tumor growth. Bone metastatic breast cancer cells show increased expression of several factors including Runx2, Gli-2, HIF-1 α and miR-218, while miR-135 and -203 levels are downregulated. Recent studies show increased autophagy in bone metastatic cells. IL-6 secretion by senescent osteoblasts promotes osteoclast differentiation and osteolysis. Inhibitory molecules and antibodies to prevent metastatic growth in bone are indicated. Anti-sclerostin antibody romosozumab also inhibits bone metastasis.

Bone metastasis is a frequent complication of cancer, with breast, prostate, lung, thyroid, and kidney cancers accounting for nearly 80% of bone metastasis. Nearly 20% of cancer patients develop detectable bone metastasis during the course of their disease while 50% show signs of metastatic disease at autopsy. Notably, among patients with metastatic breast cancer the rate of incidence for bone metastasis may be as high as 85%, with 60–75% of cases of metastatic disease initially presenting in the bone [24]. Furthermore, 79% of breast cancer patients with bone metastasis show multiple metastatic tumors in the bone. Metastatic tumors tend to develop primarily in the axial skeleton. Frequent targets of metastasis include the spine, ribs, pelvis and proximal femur. Recent studies have shown that among patients with one metastatic tumor the vertebra is most frequently affected (35%), followed by the pelvis (22%), sternum (20%), and femur (8%). While solitary metastatic tumors display a preference for the axial skeleton, among patients with multiple metastatic tumors, 53% display tumors in both the axial and appendicular skeleton vs 35% (axial) and 11% (appendicular) respectively [25,26,27,28]. Metastatic tumors within the bone can display osteolytic, osteoblastic, and mixed lesions. Osteolytic tumors account for upwards of 80% of bone metastatic tumors in breast cancer patients [29]. Metastatic tumors within the bone can contribute to complications including pathological fracture, hypercalcemia, spinal cord compression, and pain [28]. Osteolytic tumors display upregulation of PTHrP and RANKL, parathyroid hormone related protein acts via its receptor to induce a release of calcium into the circulation via bone resorption while acting in the kidney to suppress the excretion of excess calcium resulting in hypercalcemia. Furthermore, PTHrP exerts a paracrine effect on osteoblasts causing enhanced secretion of RANKL which promotes osteoclast differentiation leading to osteolysis, and liberation of insulin like growth factor (IGF) and transforming growth factor beta (TGF-β) promoting a vicious cycle of bone loss and tumor [30,31,32] (Figure 1)

Treatment options and clinical outcomes vary between subtypes of breast cancer. Breast cancer can be grouped based on morphological and molecular signatures. However, more frequently the breast cancers are classified by their molecular signature which includes potential combinations of over expression of the human epidermal growth factor receptor (HER2), progesterone (PR), and estrogen (ER) hormone receptors (HR). Breast cancers that lack these characteristics are termed triple negative [33,34,35]. Tumors expressing estrogen and progesterone receptors tend to be less aggressive compared to those over expressing HER2 or triple negative subtypes [3]. Current therapies are available to inhibit the effects of estrogen and progesterone on ER and PR positive tumors, respectively, while the monoclonal antibodies trastuzumab (Herceptin) and pertuzumab (Perjeta) function to prevent dimerization and activation of HER2 in tumors with elevated expression of HER2 [36,37]. The advent of such targeted therapies has resulted in distinct treatment options based on molecular subtype [37].

Recent studies examining the metastatic profiles of various breast cancer subtypes have shown that the bone is the most frequent site of metastasis irrespective of molecular subtype or the presence of metastatic tumors in several sites, with the brain, liver, and lung representing other preferred sites of metastasis [2,3,4,5,38]. Patients with hormone receptor positive tumors showed higher levels of bone metastasis compared against patients with triple negative, HER2+, or HER2- tumors which display visceral metastasis [3,5,27]. Similarly, a recent study of 1094 breast cancer patients exhibiting metastasis revealed that approximately 35% of patients with HR positive cancers exhibited bone only metastasis compared to 20% of HR negative and triple negative patients [39]. Examination of SEER data, which represents nearly 30% of the US population, further shows that patients with HR+ HER2- tumors account for 57.5% of bone metastatic breast cancer cases [40]. During the course of the study the proportion of patients exhibiting bone only metastasis decreased as patients tended to develop multiple metastatic tumors with the HR positive patients continuing to exhibit greater levels of metastasis when compared against HR negative. Interestingly, when examining patients with metastasis at the time of diagnoses, the bone was the most frequently observed metastatic site. In terms of survival, triple negative patients display significantly lower cancer specific survival rates relative to other breast cancer subtypes [2,24].

## 3. Regulatory Mechanisms

Previous and recent studies indicate that both tumor and tumor microenvironment-derived factors play a critical role in progression of bone metastasis via multiple mechanisms, which are discussed below.

### 3.1. Tumor-Derived Factors

#### 3.1.1. Cell Signaling

The dynamic nature of cell signaling influences the development of cancer, and the progression and severity of bone metastasis. These changes include signaling pathways such as MAPK, PI3K and RAS/RAF. A study of 40 breast cancer cell lines revealed that 25% display mutations in one or more MAPK components [41]. These mutations are typically observed in the KRAS and BRAF oncogenes resulting in uncontrolled MAPK activity. Recent studies from our group and others show that MAPK mutation-mediated dysregulation of the autophagy pathway promotes breast tumor development and bone metastasis [42,43,44]. The autophagy pathway plays a vital role in metastasis which will be discussed in further detail in this review [45].

Alterations in the transcriptional status of cancer cells has been shown to promote their tumorigenicity. Several transcription factors have been identified to promote bone metastasis such as Runx2, Hypoxia Inducible Factor 1 Alpha (HIF-1α), and Signal Transducer and Activator of Transcription 3 (STAT3) [46,47]. In this section, we reviewed the mechanisms by which Runx2 facilitates bone metastasis. The contributions of HIF-1α and STAT3 have been thoroughly reviewed by other research groups [48,49,50]. Runx2 is primarily responsible for regulating skeletal development and has been observed to be aberrantly expressed in several cancers which metastasize to the bone including breast, prostate, and lung [6,20,51]. McDonald and colleagues demonstrated an association between triple negative tumors and Runx2 expression levels. Patient stratification for Runx2 expression levels showed a poor survival rate with high Runx2 expression [52]. Aberrant expression of Runx2 in the metastatic MDA-MB-231 breast cancer cell line has been shown to promote an invasive and migratory phenotype [53]. Similarly, ectopic expression of Runx2 in MCF10A normal mammary epithelial cells and MCF-7 breast cancer cells can disrupt acini formation and promote epithelial to mesenchymal transition further contributing to tumor progression, respectively [54]. Ectopic expression of Runx2 also induces osteomimicry whereby cancer cells produce proteins typically reserved for skeletal development such as RANKL, osteoprotegrin, and matrix metalloproteinases 9 and 13, which disrupts the homeostasis of the bone microenvironment and promotes osteoclast activity while suppressing osteoblasts [55]. Serum samples from bone metastatic breast cancer patients show increased levels of sclerostin relative to both normal controls and patients with localized breast cancer. Comparable results were seen for mRNA and protein levels. The secretion of sclerostin by cancer cells is a notable finding as in its physiological context sclerostin is secreted by osteocytes to antagonize WNT signaling, resulting in suppression of bone formation [56,57]. Importantly, sclerostin antibody treatment showed 80% survival of animals with MDA-MB-231 tumor growth in the tibia with significantly higher bone mineral density (BMD), BV/TV, and trabecular thickness. Serum analysis also showed that antibody treated mice exhibit higher osteocalcin and significantly less osteoprotegrin. Interestingly, sclerostin was shown to be a transcriptional target of Runx2 and secreted by both breast cancer and myeloma cells [56,57,58,59,60]. 

Multiple transcriptional targets of Runx2 have been identified including vascular endothelial growth factor (VEGF), and HIF 1-α. These are of particular importance in the context of metastasis, in which a newly seeded metastatic tumor potentially requires the ability to induce angiogenesis to promote tumor growth. Combined, these changes in gene expression help promote the invasive and migratory phenotype associated with Runx2 expression [61]. 

Recent studies in the MDA-MB-231 model have shown that silencing of Runx2 results in impairments in the development of metastatic tumors relative to wild type cells at the early stages of bone metastasis [53]. Tan and colleagues suggest that the aberrant Runx2 expression and osteomimetic behavior observed in bone metastatic breast cancer is a consequence of epithelial to mesenchymal transition (EMT) [62]. EMT is associated with the loss of epithelial markers and cell polarity, and upregulation of mesenchymal markers such as fibronectin, collagen types I and III, and cadherin [63]. These observations further suggest that while Runx2 may be a contributor to bone metastasis it can also facilitate metastasis via EMT. 

Akech and colleagues observed elevated osteolysis in response to increased Runx2 expression using three distinct sublines of the bone metastatic prostate cancer PC3 cells which express varying levels of Runx2 [64]. A correlation between Runx2 and PTHrP expression was also observed, while co-culture of osteoclast precursor cell line RAW 264.7 RANKL and PC3-RUNX-2 high cell lines resulted in greater levels of osteoclast differentiation relative to untreated cells, or those treated with RANKL alone. These results demonstrate the interplay between cancerous cells and the bone microenvironment to promote osteolysis which can fuel the growth of a bone metastatic tumor [64,65]. 

Notch signaling represents an additional pathway which plays a role in the development of bone metastasis. The Notch receptor can be activated via its ligand Jagged 1 which has been demonstrated to be over expressed in breast cancer patients with advanced disease. Examination of the bone tropic 4T1 model of breast cancer and sub clones of MDA-MB-231 breast cancer cells that display varying levels of bone tropism revealed that highly bone tropic clones displayed the largest upregulation of Jagged 1. These findings were in agreement with patient samples that showed a significant increase in the incidence of bone metastasis in patients with high Jagged 1 expression. Interestingly, examination of Notch receptor levels did not show a correlation with disease. These results were further confirmed with animal studies showing that over expression of Jagged 1 enhanced the development of bone metastatic tumors in mice while silencing of Jagged 1 delayed the onset of metastasis [66].

The activation of Notch via tumor derived Jagged 1 induces the production of IL-6 by osteoblasts which has a positive effect on osteoclast differentiation and allows for the initiation of the vicious cycle of osteolysis and tumor development. Furthermore, Jagged 1 serves as an effector of the TGFβ pathway which has been known to play a role in the development of bone metastasis as osteolysis liberates TGFβ from the bone matrix [66].

#### 3.1.2. Tumor Microenvironment Derived Factors

Tumor Mediated Alteration of the Bone Microenvironment: Among the most significant consequences of metastasis is the damage induced to the surrounding tissue. In the context of breast cancer metastasis to the bone, patients often present with osteolytic lesions to the bone. The interaction between the tumor and the bone microenvironment causes the balance between localized deposition and resorption of bone to shift towards resorption. While the initial shift is often induced by factors secreted by tumor cells such as RANKL and PTHrP, the resulting degradation of bone releases a substantial supply of insulin like growth factors I and II (IGF-I, IGF-II), TGF-β, platelet derived growth factor (PDGF), and fibroblast growth factor (FGF) along with bone morphogenetic proteins (BMP) and other factors from the ECM which in turn promote tumor growth and development. This phenomenon termed the “vicious cycle” results in bone loss, increased risk of fracture, hypercalcemia, and spinal cord compression and represents a significant cause of patient disability and mortality. Among cancer patients the most frequent sites of pathological fractures include the long bones, particularly the femur, as well as the ribs and vertebrae [6,7,29,64,65]. In addition to promoting bone resorption, tumor cells have also been demonstrated to suppress osteoblast differentiation while promoting the development of osteoclasts, further intensifying the damage to the bone surrounding a metastatic tumor [55]. Recent studies examining tumor mediated suppression of osteoblast differentiation have shown that tumor cells secrete the WNT antagonist sclerostin. Treatment of bone marrow derived mesenchymal stem cells and MC3T3 cells with conditioned media from MDA-231 breast cancer cells suppressed osteoblast differentiation, while conditioned media pretreated with anti-sclerostin antibody attenuated the suppression of differentiation [57]. PTHrP and parathyroid hormone serve to initiate the cycle by binding to their corresponding receptor causing an induction of RANKL. RANKL serves as ligand to the receptor activator of nuclear factor kappa B (RANK) receptor on osteoclast progenitor cells. The interaction between ligand and receptor serves to promote osteoclast differentiation. Interestingly, TGF-β released through degradation of the bone results in the upregulation of PTHrP by cancer cells further perpetuating the cycle. Nearly 92% of bone metastatic breast cancers express PTHrP compared to approximately 50% of primary cases. In addition to PTHrP other factors such as IL-6, IL-1, prostaglandin E2, macrophage colony stimulating factor, and tumor necrosis factor alpha (TNF-α) have been shown to contribute to osteoclastogenesis. Prostaglandin E2 in particular can directly cause increases in RANKL while also enhancing its effects on osteoclastogenesis [7,65,67,68].

Growth factors liberated from the bone via osteolysis have the potential to enhance metastasis and tumor development. Recently, it was shown that silencing of Runx2 in MDA-231 breast cancer cells results in delayed metastasis and late stage tumor development. Interestingly, upon examination of parental cells, lung metastasis and bone metastasis derived cells, bone derived Runx2 knockdowns displayed significantly upregulated IGF-1Rβ expression while parental and lung derived cells showed no difference in expression with knockdown. In addition to increased receptor expression bone derived knockdowns displayed enhanced AKT activity in response to IGF-1 stimulation which was completely suppressed with chemical inhibition of IGFR but not PI3K or MEK-ERK 1/2. These results were recapitulated in ex vivo bone co cultures and bone derived PC3 prostate cancer cells. These results highlight the importance of IGFR signaling in bone metastasis and the role of the microenvironment in promoting tumor development and progression [53].

The CXCR4-CXCL12 stromal cell derived factor-1(SDF-1) axis represents another factor in the local microenvironment which has been shown to influence the metastatic process. Osteoblasts and bone marrow stromal cells display elevated expression of SDF-1 which serves to regulate the homing of hematopoietic stem cells to the bone marrow. Recent studies have shown that several cancer types including breast and prostate overexpress the corresponding receptor CXCR4 providing a mechanism for the homing of tumor cells to the bone microenvironment. Interestingly, activation of the SDF-1 CXCR4 axis results in tumor mediated secretion of CXCL16 to recruit CXCR6 positive mesenchymal stem cells to the tumor microenvironment. Recruited mesenchymal stem cells in turn become cancer associated fibroblasts which secrete high levels of SDF-1 to perpetuate the cycle of tumor cell homing and development [21,30]. Recent studies using the bone metastatic MDA-231 breast cancer cell line have shown that CXCR4 was over expressed in single cell clones which metastasize to the bone relative to the parental population. Interestingly, CXCR4 over expression was not observed in lung metastatic clones, highlighting the heterogeneity within a primary tumor [21]. These observations were recapitulated using an in vivo passaging model to generate a highly bone metastatic clone of MDA-231, with CXCR4 representing one of the most overexpressed genes within the population. Similarly, over expression of CXCR4 in the parental population increased the rate of bone metastasis in animal models [69].

## 4. Regulatory Pathways

Several cellular pathways have been shown to be altered in metastatic breast cancer cells resulting in enhanced bone metastasis. Recently, the autophagy pathway and microRNAs have been shown to facilitate bone metastasis. These reports are discussed below. 

### 4.1. Autophagy Dysregulation and Metastasis

Autophagy, or self-digestion, is a highly selective cellular pathway involved in protein and organelle degradation. Autophagosomes fuse with lysosomes and degrade contents into macromolecular subunits which can then be recycled into various metabolic pathways. Basal levels of autophagy maintain cellular homeostasis. Under normal circumstances autophagy is strictly regulated by the AMPK, mTOR, and PI3K pathways with AMPK and mTOR serving as positive and negative regulators, respectively. MAPK mutations abolish this regulation resulting in highly active autophagy pathway, rather than a basal level which can be upregulated under conditions such as metabolic or hypoxic stress [42,44,70]. 

Recent studies utilizing breast cancer cell lines with MAPK mutations or expressing mutant RAS produced several key findings which suggest that MAPK mutation contribute to autophagy dysregulation and show a similar phenotype to that of aberrant Runx2 expression. During metastasis, tumor cells detach from the extracellular matrix (ECM) and risk an apoptotic cell death known as anoikis [71]. MAPK mutations are not frequently observed in breast cancer, a survey of commonly used breast cancer cell lines reported that 25% (10) of cell lines surveyed contained mutations in KRAS, BRAF, HRAS, or NRAS, with two cell lines displaying more than one mutation [41]. Human breast cancer cell lines display varying degree of metastatic potential. Intra-iliac artery injection can be utilized as a method for studying micrometastasis which can model early stage disease, while intra-cardiac and orthotopic implant methods allow for examination of disease progression [72,73]. The intra-caudal arterial injection has been shown to deliver breast cancer cells to the bone marrow of hind limbs with higher efficiency than intra-cardiac injection. [74]. MDA-MB-231 cells are frequently used to model bone metastasis as they can produce osteolytic tumors in bone following orthotopic or intracardiac inoculation and produce an osteolytic phenotype reminiscent of that observed in patients [73]. MDA-MB-231 display mutations in both KRAS and BRAF, the work summarized below utilizes a combination of MDA-MB-231 and transformed MCF-10A cells to dissect the contributions of autophagy to breast cancer pathogenesis [41]. 

Recent reports have demonstrated that induction of autophagy served a role in helping cells resist anoikis [71,75]. Using mammary epithelial MCF-10A cells grown under both adherent and suspension culture conditions, Fung and colleagues demonstrated a robust induction of autophagy under detached conditions. In terms of functional consequences a series of cells in which key autophagy related genes (ATG) -5,-6, and -7 were silenced resulted in loss of autophagy induction, increased caspase-dependent cell death, and impaired re-plating efficiency upon transfer of suspended cells to adherent culture conditions [71,76]. Lock and colleagues utilized both RAS mutant cell lines in conjunction with cells virally transformed to express mutant RAS and demonstrated significantly elevated levels of colony formation in soft agar assays in response to mutated RAS, which was attenuated when ATG’s 5 or 7 were silenced. These results demonstrate that autophagy dysregulation can overcome anoikis and potentially contribute to metastatic potential [77]. Mori and colleagues were able to identify an anchorage independent growth gene signature through the use of a series of 19 anchorage dependent and independent breast cancer cell lines and DNA micro array studies. Clinically, these studies offer a potential means of screening for a given tumors metastatic potential and may help clinicians identify patients whose cancer may metastasize prior to the development of metastatic tumors [78]. While resistance to anoikis enhances the metastatic potential, cells must also be able to migrate to a metastatic niche upon detachment. Among the phenotypes displayed by cancers with dysregulated autophagy is that of increased migration. Studies utilizing MDA-MB-231 cells, which carry both KRAS and BRAF mutations, showed significant decreases in cellular migration in response to genetic silencing of ATG’s 7 or 12 as well as chemical inhibition of autophagy with Bafilomycin A1. These results were supported through the use of an in vivo metastasis assay which revealed that silencing of ATG 7 or 12 reduced the ability of HRAS mutant MCF-10A cells to produce lung metastases [79]. Upon further examination Lock and colleagues demonstrated that expression of mutant HRAS in MCF-10 cells caused a significant increase in IL-6, promoting cellular migration and invasion. Levels of secreted IL-6 were significantly decreased following silencing of ATG 7 or 12 [79]. The identification of autophagy mediated IL-6 secretion was notable in the context of bone metastasis, as IL-6 was shown to be secreted by senescent osteoblasts, serving to promote osteoclast differentiation, resulting in enhanced osteolysis and tumor burden in mouse models. Ex vivo models of osteoclast differentiation displayed suppression of differentiation in the presence of IL-6 antibody [80,81]. These results highlight the dynamic relationship between tumor cells and resident cells of the bone microenvironment and emphasize the contributions of tumor autophagy to shifting the balance to favor tumor seeding and progression. 

**Figure 2:**
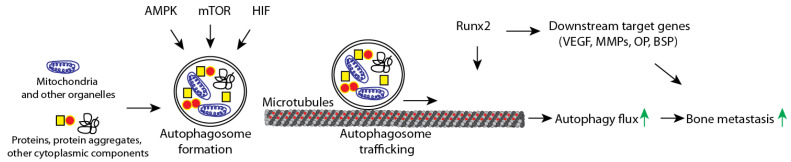
Autophagy is positively regulated by AMPK and HIF signaling pathways with the mTOR pathway serving as a negative regulator. Upon induction of autophagy damaged organelles and macromolecules are engulfed in a double membrane vesicle known as an autophagosome. Autophagosomes traffic along microtubules towards the lysosome to allow for fusion and enzymatic degradation of autophagosomes and their contents into macromolecular subunits (flux). Runx2 serves a dual role in promoting metastasis, (1) through the enhancement of microtubule acetylation and autophagosome trafficking, and (2) providing transcriptional support and upregulating genes implicated in bone metastasis such as VEGEF, assorted MMP’s, osteopontin (OPN), and bone sialoprotein (BSP), among others.

Similar to autophagy, dysregulation via Runx2 has also been demonstrated to promote invasion and migration. Wound healing assays revealed significantly less migration of MDA-MB-231 cells following silencing of Runx2. Conversely, over expression of Runx2 in H-1299 lung cancer cells caused significant increases in wound healing in response to TGF-β treatment [82,83]. The results from studies in the MDA-MB-231 model are of particular importance as they both express Runx2 and mutations in KRAS and BRAF. More recently, we have elucidated an interaction between Runx2 and the autophagy pathway [42]. Using bone-derived isogenic variants of control and Runx2 knockdown MDA-MB-231 cells we found that Runx2 is required for autophagy. Runx2 silencing arrests the autophagy pathway resulting in the lack of degradation of autophagic vesicles [42]. Interestingly, comparison of autophagy among parental and bone-derived breast cancer cells revealed elevated autophagy among bone-derived control cells relative to their parental counterparts [42]. Further analysis demonstrated a decrease in α-Tubulin acetylation with Runx2 silencing. Acetylated microtubules serve as a conduit for autophagic vesicles destined for lysosomal degradation [42]. These findings were of note as high levels of Runx2, autophagy dysregulation, and elevated α-tubulin acetylation have each been independently linked with a phenotype of invasive and migratory cancer. These studies suggest that Runx2 can enhance trafficking of autophagosomes via tubulin acetylation and subsequently lead to increased cell survival during bone metastasis. Taken together, these studies suggest that crosstalk between cell signaling pathways and Runx2 could lead to invasive cellular behavior that may contribute to the bone metastasis of breast cancer (Figure 2).

Recently, Rab5a has been shown to regulate autophagy in bone tropic MDA-MB-231 cells. During the early stages of autophagy Rab5a interacts with Beclin1 to promote endosome and autophagosome formation. Rab5a also interacts with ATG7 in the terminal stages to promote lipidation of LC3. Lipidated LC3 is incorporated into the autophagosome membrane and plays a role in both the formation of the autophagosome and recruitment of cargo for subsequent degradation [84]. Further studies show an increase in Rab5a incorporation into endosomal and lysosomal compartments in response to amino acid starvation, where it plays a role in regulating the activity of mTORC1 and the autophagy pathway [85]. Interestingly, examination of parental vs a bone tropic clone of MDA-MB-231 cells showed a greater than 3 fold upregulation of Rab5a in bone tropic cells. Histological analysis of patient specimens showed progressive enhancement of Rab5a signal between normal mammary tissue and mid and high grade dysplasia. Bone metastatic tumors showed high levels of staining with both nuclear and cytoplasmic localization [84]. While survival data was not available in the prior study, a recent study found significantly elevated levels of transcription factor EB (TFEB) protein in pancreatic cancer specimens relative to adjacent healthy tissue. High levels of TFEB correlated with decreased survival. In addition to elevated TFEB expression the authors observed enhanced TFEB- dependent transcription of Rab5a, with elevated Rab5a expression in patient samples resulting in significantly decreased overall survival. This interaction is notable as TFEB serves as a master regulator of lysosome biogenesis and a central regulator of the autophagy process [86].

### 4.2. Micro RNA and Metastasis

microRNAs (miR) contribute to a variety of malignancies. Changes in the expression levels of several miRNAs have been reported in breast cancer, with miR 218, 218-5P, 135, 203, and 34a-5P being notable in the context of bone metastasis [87,88,89,90,91]. 

Breast cancer cell lines and patient-derived bone metastatic samples showed diminished expression of miR 135 and 203, while expression was significantly higher in MCF-10A normal mammary epithelial cells and non-metastatic MCF-7 breast cancer cells [88]. Transfection of miR 135 and 203 mimetics reduces wound healing, transwell migration, and proliferation of MDA-231 cells. These results were recapitulated in animal models which showed a significant reduction in spontaneous bone metastasis of mammary fat pad tumors generated from MDA-231 cells ectopically expressing either miRNA combined with intratumoral injection of synthetic miRNA oligonucleotide. Further studies using an intra tibial model yielded similar findings in addition to reduced osteolysis and TRAP positive osteoclasts at the bone tumor interface. It should be noted that a reciprocal relationship between miRNA expression and Runx2 was observed highlighting the interactions between multiple pathways discussed in this review and the development of bone metastasis [88].

In contrast, miR 218 and 218-5P were shown to have pro-tumorigenic effects [89,90]. High levels of miR 218-5P have been reported in bone metastasis patient samples compared to primary tumor samples. Intratibial injection of MDA-MB-231 breast cancer cells over expressing miR 218-5P showed significant increases in tumor size, osteolysis, and proliferation which were attenuated in cells expressing anti miR218-5P oligonucleotides. Mechanistically, miR 218-5P was shown to promote WNT Signaling through the suppression of WNT antagonists sclerostin (SOST) and secreted frizzled related protein 2 (sFRP-2), while promoting the expression of pro metastatic genes CXCR4, bone sialoprotein (BSP), osteopontin (OPN), and PTHRP [89]. Table 1 summarizes the tumor and bone-derived factors contributing to bone metastasis of breast cancer.

**Table 1:** Factors contributing to bone metastasis.

Factor	Functional Description	Reference
Signaling/Secreted Factors		
ADM	Potentiates osteolytic responses in bone to metastatic breast cancer	[92]
ANGPTL2	Up-regulates CXCR4 expression in tumor cells, enhancing responsiveness of breast cancer cells to bone tissues secreting CXCL12	[93]
DKK1	Promotes bone metastasis by regulating canonical WNT signaling of osteoblasts	[94]
IL-1β	Stimulates breast cancer colonization by inducing NFkB/CREB-Wnt signaling	[95]
IL-6	Inhibition of IL-6 reduces MDA-231 bone metastasis by inhibiting cell proliferation and decreasing expression of P-Stat3, VEGF, and RANK	[96]
IL-11	Promotes bone metastasis through JAK1/STAT3 signaling pathway in BoM-1833 cells	[97]
ITGBL1	Facilitates recruitment, residence, and growth of breast cancer in bone	[98]
Jagged1	Tumor-derived ligand that activates Notch signaling in bone, promoting IL-6 secretion and subsequent osteolytic bone metastasis	[63,99]
OPN	Enhances ability of CD44+ breast cancer cells to migrate to the bone, potentially through activation of WINK-1 and PRAS40-related pathways	[100]
Sclerostin	Promotes cell migration, invasion, and bone osteolysis	[60]
VCAM-1	Promotes metastasis by interacting and recruiting α4β1-positive osteoclast progenitors	[101]
Transcription Factors		
HIF-1α	Promotes metastatic spread by upregulating PTGS2/COX-2 and by increasing expression of CXCL12 in osteoprogenitor cells	[102,103]
Runx2	An essential regulator of skeletal development, Runx2 is highly expressed in breast cancer skeletal metastases, and is associated with tumor-induced osteolysis. Runx2 activity is promoted via the PI3K/AKT signaling pathway. Evidence shows that expression in bone metastasis is regulated by miR-135 and miR-203. Functions also in increasing cell proliferation via disrupting growth-arresting acini structures, and promoting cell survival by enhancing the autophagic process.	[20,42,48,49,50,51,52,54,58,59,85]
GLI-2	Increases secretion of osteolytic factors such as parathyroid hormone-related protein (PTHrP)	[46,47]
STAT3	Contributes to migration, invasion, and angiogenesis	[104]
Micro RNA		
miRNA 135 miRNA 203	Both show diminished expression in bone metastatic MDA-231 cells, and are related to aberrant expression of Runx2	[88]
miRNA 218-5P	Highly expressed in bone metastatic breast cancer cells, functioning to promote WNT signaling and enhance osteolysis	[89]
Enzymatic Proteins		
ADAMTS1 MMP1	Modulate bone microenvironment in favor of osteoclastogenesis and bone metastasis	[105]
MMP13	Upregulation contributes to osteoclast differentiation by activating MMP-9 and promoting cleavage of galectin-3	[106]
MFAP5	Increases and accelerates bone metastasis, possibly by increasing expression of MMP2, MMP9, and activating the ERK signaling pathway	[107]
Receptors and Growth Factors		
βAR	βAR stimulation in osteoblasts may activate bone marrow vessels to favor skeletal engraftment of breast cancer cells	[108]
BMPR1a	BMPR1a promotes osteolytic metastasis of breast cancer cells by promoting RANKL production via the p38 pathway	[109]
FGFR1/FGF	FGFR1 activation by tumor cell-derived FGF ligands enhance osteoclast function, contributing to metastatic lesions	[110]
Notch	Activation of Notch signaling in bone microenvironment via tumor-derived Jagged1 promotes osteoclast differentiation and facilitates metastasis by initiation EMT. Activation via tumor-derived Galectin-3 has been shown to inhibit osteoblast differentiation	[63,99,111]
IGF-1R/IGF	IGF-1R activation by bone-derived IGF stimulates bone metastasis via activation of the Akt/NF-κB signaling pathway	[112]
C-Met/HGF	C-Met and cognate ligand HGF expression in bone correlates with bone metastasis	[113]
RANK/RANKL	RANKL expression and RANK activation is induced by tumor-derived factors, promoting osteoclast activity and increasing bone invasiveness	[114]
TGF-β	TGF-β released in bone matrix upon tumor-induced osteolysis further stimulates bone metastatic cells to secrete factors driving osteolytic bone destruction	[115]
VEGF	VEGF is expressed strongly in breast cancer metastases, and in the presence of RANKL can stimulate formation of osteoclasts	[116]
Autophagy		
ATG7 ATG12	ATG7 and ATG12 inhibition in MDA-231 cells resulted in decreased colony formation and proliferation	[77]
Rab5a	Rab5a expression is elevated in metastasized 1833 cells, and may be related to cell survival by triggering autophagy and autophagosome sealing	[84]
Chemokines		
COX-2/CXCR2	CXCR2 enhances breast cancer metastasis to bone by suppressing AKT1 and activating COX-2	[117]
CXCL10/CXCR3	CXCL10/CXCR3 axis contributes to breast cancer metastasis and osteoclast activation in 4T1 cells	[118]
CXCL12/CXCR4	CXCL12/CXCR4 axis promotes breast cancer metastasis to tissues expressing high levels of CXCL12	[119]

Tumor and bone-derived factors contributing to bone metastasis of breast cancer cells. Abbreviations: Signaling and Secreted Factors: ADM (adrenomedullin), ANGPTL2 (angiopoietin-related protein 2), DKK1 (Dickkopf-related protein 1), IL (interleukin), ITGBL1 (integrin subunit beta like 1), OPN (osteopontin), VCAM1 (vascular cell adhesion protein 1). Transcription Factors: HIF-1α (hypoxia-inducible factor), Runx2 (runt-related transcription factor 2), STAT3 (signal transducer and activator of transcription 3). Enzymatic Proteins: ADAMTS1 (a disintegrin and metalloproteinase with thrombospondin motifs 1), MMP (matrix metalloproteinase), MFAP5 (microfibrillar-associated protein 5). Receptors and Growth Factors: βAR (beta adrenergic receptor), BMPR1a (bone morphogenetic protein receptor type 1a), FGF (fibroblast growth factor), FGFR1 (FGF receptor 1), IGF (insulin-like growth factor), IGF-1R (IGF 1 receptor), C-Met (c-Met tyrosine kinase receptor), HGF (hepatocyte growth factor), RANK (receptor activator of nuclear factor κ B), RANKL (RANK ligand), TGF- β (transforming growth factor beta), VEGF (vascular endothelial growth factor). Autophagy: ATG (autophagy related), Rab5a (Ras-related protein Rab-5a). Chemokines: COX-2 (cyclooxygenase-2), CXCL (C-X-C motif chemokine ligand), CXCR (C-X-C motif chemokine receptor).

## 5. Metastatic Bone Disease: Clinical Management and Perspectives

### 5.1. Therapeutic Approaches to Managing Metastatic Bone Disease

Pathologic fractures can occur due to altered biomechanics and weakness in bone due to a lesion secondary to MBD. One study found that 4.4% of nearly 300,000 hospital admissions associated with bone metastases had a primary diagnosis of pathologic fracture [120]. Various risk factors for pathologic fracture have been identified in numerous studies; however, commonly found risk factors include age, menopausal status, osteoporosis at time of bone metastasis, and hormone receptor status [121]. Scoring systems have been established to determine risk of impending fracture utilizing factors such as site of lesion, nature of lesion, whether lytic or blastic, extent of lesion, and pain [122,123].

In the setting of high risk or impending fracture in MBD, prophylactic stabilization should be considered to prevent these events. The benefits of surgical fixation of pathologic fracture are well documented. One large National Inpatient Sample (NIS) study found surgical fixation to be associated with increased survival in pathologic fractures [124]. Other studies have shown improved ability for ambulation, pain relief, and increased stability with surgical treatment [125,126]. Although it has been shown that treatment of these realized pathologic fractures is beneficial, significant literature has also shown evidence of the benefit in treating impending fractures. Approaches to prophylactic stabilization include intramedullary nail fixation [127,128,129], arthroplasty [130], and plating with potential use of cement [131,132].

Large database studies found prophylactic stabilization to be associated with lower odds of adverse events, death, blood transfusion, increased length of hospital stay, and decreased mortality and treatment cost compared to fracture fixation [133,134]. An institutional study found prophylaxis to be associated with less average blood loss, shorter hospital stays, higher likelihood of discharge to home vs. extended care facility, and greater likelihood of support-free ambulation [135]. Other studies have also reported prophylaxis to have improved survival compared to fracture fixation [136,137]. 

Surgical treatment of pathologic fractures can improve pain and function as well as quality of life; however, there are inherent problems associated with treatment. Treatment failure rates requiring reoperation have been reported by several studies. A 2016 review by Janssen et al. investigated 40 studies on the topic and reported reoperation rates ranging from 0–31% after endoprosthetic reconstruction, 0–26% after intramedullary nailing, and 0–42% after open reduction-internal fixation. One study identified pathologic fracture as an independent risk factor for intramedullary nail breakage in proximal femoral fractures [138]. Another study showed a similar increased risk of nail failure associated with pathologic fracture as well as preoperative radiotherapy [139]. Reoperation is associated with its own risks; one study found a near 10% deep prosthetic infection rate in patients undergoing hip arthroplasty for failed treatment of pathologic fracture [140].

### 5.2. Current Challenges and Future Directions

Current therapeutic options for patients exhibiting bone metastasis emphasize the need to maintain bone quality and prevent the further destruction of the surrounding bone. Currently bisphosphonates are among the most widely utilized agents. Clinically, bisphosphonates are prescribed for patients with Multiple Myeloma, and solid tumors of the breast and prostate [67]. Bisphosphonates are analogs of pyrophosphate and display high affinity for mineralized bone generating elevated levels of bisphosphonate in regions of bone exposed due to resorption. The internalization of bisphosphonate by osteoclasts causes impairment of the resorption process and has been shown to induce apoptosis of osteoclasts thereby preventing further resorption [68]. Currently zoledronic acid a third generation bisphosphonate with 100 times greater efficacy relative to the 1st generation bisphosphonate pamidronate has been approved for use in patients with multiple myeloma, lung, prostate, and breast cancer patients with bone metastasis. A recent meta-analysis which examined the results of 18 randomized controlled trials containing a sample size of 5600 patients revealed that among women with advanced breast cancer and evident metastasis the use of bisphosphonates significantly reduced the risk of skeletal related events (excluding hypercalcemia) by 15%, while also delaying the onset of skeletal related events by 3–6 months. However, overall survival and the incidence of new metastases were unchanged in patients with locally advanced or metastatic breast cancer [141]. In addition to bisphosphonates, the monoclonal antibody denosumab against RANKL has shown efficacy in preventing further resorption of bone via curbing osteoclastogenesis. It should be noted that while showing a similar side effect profile to bisphosphonates which includes nausea, diarrhea, and potentially osteonecrosis of the jaw, denosumab does not accumulate in bone tissue which renders these effects potentially reversible upon discontinuation of treatment [29]. Recent studies comparing the efficacy of denosumab against that of zoledronic acid in breast cancer patients with bone metastasis show a significant delay of 18% in the onset of the first skeletal related event. Additionally among patients receiving denosumab there were significant reductions in the risk of developing multiple skeletal related events (23%), skeletal morbidity rate (22%), and bone turnover markers N-terminal telopeptide (uNTX) 80% and bone specific alkaline phosphatase (BSAP) 40% [142]. While bisphosphonates and denosumab represent the standard options for the treatment of tumor induced osteolytic bone disease, clinical trials are in progress examining the efficacy of autophagy inhibition in curbing tumor development and potentially metastasis. Furthermore, inhibitors of key autophagy regulators PI3KC3, ATG4B, and ATG7 are being developed for potential use in clinical trials [143,144]. While results from clinical trials are currently unavailable preclinical studies suggest that the use of autophagy inhibitors such as chloroquine may potentially curb tumor growth and curb metastasis. 

Other proposed therapeutic targets include Sclerostin. While the anti-sclerostin antibody romosozumab has yet to be evaluated for patients with bone metastasis, existing data from preliminary studies suggest a benefit for patients [58,59,60]. Recent studies aimed at targeting the transcriptional component of cancer development have yielded a small molecule inhibitor of Runx2, CADD522. Treatment of breast cancer cells with CADD522 reduced viability and clonogenicity while also suppressing transcriptional activity of Runx2. Animal models yielded decreased tumor weight, volume, and incidence, and onset with treatment in a dose dependent manner [145]. The role of TGFβ in promoting tumor development and metastasis, combined with the liberation of TGFβ during periods of tumor induced osteolysis has been well documented, leading to the examination of TGFβ inhibition via soluble decoy receptors, small molecule inhibitors, and antisense oligonucleotides as a potential therapeutic target for patients with bone metastasis [115]. Several recent pre-clinical models have shown the efficacy TGFβ inhibition via the pan TGFβ antibody 1D111 or the chemical inhibitor LY2109761. Antibody treatment resulted in significant decreases in osteolysis and tartarate resistant acid phosphatase (TRAP) positive osteoclasts at the bone-tumor interface. Antibody treatment also resulted in increased survival and decreased metastatic tumor burden in bones [146,147]. 

While progress has been made elucidating mechanisms of bone metastasis and treatment of tumor induced osteolysis, bone metastasis remains a significant contributor to patient mortality and a challenge for clinicians. Recent basic science and pre-clinical studies offer the potential for the establishment of new biomarkers such as LC3B, p62, and Runx2, and treatments such as sclerostin antibody, miRNAs targeting Runx2, small molecule inhibitors against cell signaling may show some potential that help curb the downstream effects of gene expression in tumors or bone microenvironment which may be shifting the balance towards metastasis. 

## 6. Conclusions

Despite recent advances in our understanding of the process of cancer metastasis and treatment options, metastatic bone disease remain a leading cause of cancer-related deaths. In this review, we summarized the research findings that revealed how metastatic cancer cells alter the activity of osteoclasts and osteoblasts, consequently promoting tumor growth, severe bone loss, and fracture. Cell signaling pathways and secretory factors including TGF-β, IGF-1, Notch, IL6, CXCR4, and PTHrP contribute to tumor growth in the bone microenvironment. Recent studies indicate that growth factor signaling pathways, secretory factors, miRNAs, and regulatory factors such as Runx2 are promising targets in reducing bone metastatic tumor growth and bone loss in preclinical models. The major challenges in reducing patient mortality associated with bone metastasis include a lack of understanding the early survival mechanisms of metastatic cancer cells in the bone microenvironment, changes in the tumor microenvironment, efficient animal models, and targeted therapies. Novel mouse models of bone metastasis such as intra-iliac artery and intra-caudal artery injections of human breast cancer cells can help define the mechanisms of early stages of bone metastasis. Recent studies showing upregulation of the autophagy pathway contributing to early survival of bone metastatic cells may further reveal novel therapeutic targets. Several basic and preclinical studies showing the potential for establishing new biomarkers such as autophagy pathway proteins and Runx2, as well as treatments such as a sclerostin antibody, miRNAs, and the combination of small molecule inhibitors against cell signaling may inhibit the downstream effects of metastasis-related molecular changes in tumors or bone microenvironment.
